# Identification of genetic and environmental factors influencing aerial root traits that support biological nitrogen fixation in sorghum

**DOI:** 10.1093/g3journal/jkad285

**Published:** 2023-12-14

**Authors:** Emily S A Wolf, Saddie Vela, Jennifer Wilker, Alyssa Davis, Madalen Robert, Valentina Infante, Rafael E Venado, Cătălin Voiniciuc, Jean-Michel Ané, Wilfred Vermerris

**Affiliations:** Plant Molecular and Cellular Biology Graduate Program, University of Florida, Gainesville, FL 32609, USA; Plant Molecular and Cellular Biology Graduate Program, University of Florida, Gainesville, FL 32609, USA; Department of Bacteriology, University of Wisconsin, Madison, WI 53706, USA; Department of Microbiology and Cell Science, University of Florida, Gainesville, FL 32610, USA; Independent Junior Research Group–Designer Glycans, Leibniz Institute of Plant Biochemistry, 06120 Halle (Saale), Germany; Department of Horticultural Sciences, University of Florida, Gainesville, FL 32609, USA; Department of Bacteriology, University of Wisconsin, Madison, WI 53706, USA; Department of Bacteriology, University of Wisconsin, Madison, WI 53706, USA; Department of Horticultural Sciences, University of Florida, Gainesville, FL 32609, USA; Department of Bacteriology, University of Wisconsin, Madison, WI 53706, USA; Department of Agronomy, University of Wisconsin, Madison, WI 53706, USA; Department of Microbiology and Cell Science, University of Florida, Gainesville, FL 32610, USA; University of Florida Genetics Institute, University of Florida, Gainesville, FL 32610, USA

**Keywords:** adventitious root, bioenergy, brace root, diazotroph, GWAS, minicore, mucilage, nodal root, polysaccharide, *Sorghum bicolor* (L ) Moench

## Abstract

Plant breeding and genetics play a major role in the adaptation of plants to meet human needs. The current requirement to make agriculture more sustainable can be partly met by a greater reliance on biological nitrogen fixation by symbiotic diazotrophic microorganisms that provide crop plants with ammonium. Select accessions of the cereal crop sorghum (*Sorghum bicolor* (L.) Moench) form mucilage-producing aerial roots that harbor nitrogen-fixing bacteria. Breeding programs aimed at developing sorghum varieties that support diazotrophs will benefit from a detailed understanding of the genetic and environmental factors contributing to aerial root formation. A genome-wide association study of the sorghum minicore, a collection of 242 landraces, and 30 accessions from the sorghum association panel was conducted in Florida and Wisconsin and under 2 fertilizer treatments to identify loci associated with the number of nodes with aerial roots and aerial root diameter. Sequence variation in genes encoding transcription factors that control phytohormone signaling and root system architecture showed significant associations with these traits. In addition, the location had a significant effect on the phenotypes. Concurrently, we developed F_2_ populations from crosses between bioenergy sorghums and a landrace that produced extensive aerial roots to evaluate the mode of inheritance of the loci identified by the genome-wide association study. Furthermore, the mucilage collected from aerial roots contained polysaccharides rich in galactose, arabinose, and fucose, whose composition displayed minimal variation among 10 genotypes and 2 fertilizer treatments. These combined results support the development of sorghums with the ability to acquire nitrogen via biological nitrogen fixation.

## Teaser

Select accessions of the cereal crop sorghum form mucilage-producing adventitious nodal roots that harbor nitrogen-fixing bacteria, which could contribute to agricultural sustainability by reducing the reliance on chemical fertilizers. Sorghum is gaining global interest as a versatile and climate-resilient crop. Wolf et al. report an effort to determine the genetic and environmental factors contributing to the formation of these so-called aerial roots as the basis for breeding programs aimed at sustainable production. Despite considerable phenotypic variation, the authors identify loci associated with the number of nodes with aerial roots and aerial root diameter.

## Introduction

Plant breeding and genetics have played a major role in the adaptation of plants to meet human needs since the dawn of agriculture some 10,000 years ago, starting with the selection of individual plants that would not disperse their seeds (De Wet 1975). More recently, the selection of alleles conferring photoperiod-insensitivity enabled the cultivation of crop plants at higher latitudes (Maheswaran et al. 2000; Coles et al. 2010; Morris et al. 2013). The Green Revolution in the 1950s and 1960s enabled significant increases in grain yield via the selection of alleles that reduced plant height and leaf angle, resulting in cereals with a greater harvest index (Russell 1985; Hay 1995; Reynolds et al. 1999; Multani et al. 2003; Yamaguchi et al. 2016). The Green Revolution also generated a dependency on synthetic fertilizer, which had become abundantly available as a result of the industrial production of ammonia via the Haber–Bosch process (Peoples et al. 1995; Houlton et al. 2013). Agriculture's current challenge is to sustainably meet the demand for food, feed, fodder, fiber, and renewable chemicals and fuels of the growing world population (Arora 2019). Reducing the use of synthetic fertilizer for crop production will lower the current 2.1% contribution (1.3 × 10^9^ Mt CO_2_eq per year) of greenhouse gases from the Haber–Bosch process (Menegat et al. 2022). Another benefit is a reduction in nitrogen fertilizer runoff, limiting soil acidification (Schroder et al. 2011), and nitrate contamination of groundwater and surface water (Kellman and Hillaire-Marcel 2003).

Biological nitrogen fixation (BNF) offers a sustainable mechanism for providing crops with nitrogen while preserving crop quality and yields without the (over)use of fertilizer. Diazotrophic microorganisms can establish symbiotic associations with select plant species and reduce nitrogen (N_2_) via a nitrogenase metalloenzyme complex to generate ammonium (NH_4_^+^) in exchange for a carbon source (Burgess and Lowe 1996). This symbiotic relationship has been best studied in legumes where rhizobia colonize root nodules, providing an environment with low oxygen pressure to enable nitrogenase activity. The rhizobia supply 50 to 80% of the nitrogen required for plant growth (reviewed by Yun et al. (2023)). Although diazotrophs have been reported to fix nitrogen on cereal crops through endophytic or rhizospheric associations, the amount of nitrogen fixed is often variable and generally low relative to legumes (Gantar et al. 1991; Riggs et al. 2001; Estrada et al. 2002; Hurek et al. 2002; Bertalan et al. 2009; Barros et al. 2020).

Indigenous maize (*Zea mays* L.) landraces from Totontepec Villa de Morelos in the Sierra Mixe region of Oaxaca, Mexico, have been cultivated by Mixe farmers for presumably hundreds to thousands of years to grow in nutrient-poor soil. The Sierra Mixe maize has been reported to obtain 29 to 82% of its nitrogen needs through BNF (Van Deynze et al. 2018). The Sierra Mixe maize is characterized by the formation of aerial roots on as many as 10 stem nodes that produce mucilage after rain (Pankievicz et al. 2022). Aerial roots are adventitious nodal roots that, unlike brace roots, do not make contact with the soil. The mucilage associated with aerial roots provides a sugar-rich, hypoxic environment to host a nitrogen-fixing microbiome (Osborn et al. 1999; Pozzo et al. 2018; Van Deynze et al. 2018). These findings in maize serve as the basis for exploring nitrogen-fixation activity in other cereal crops that form aerial roots.

**Fig. 1.**
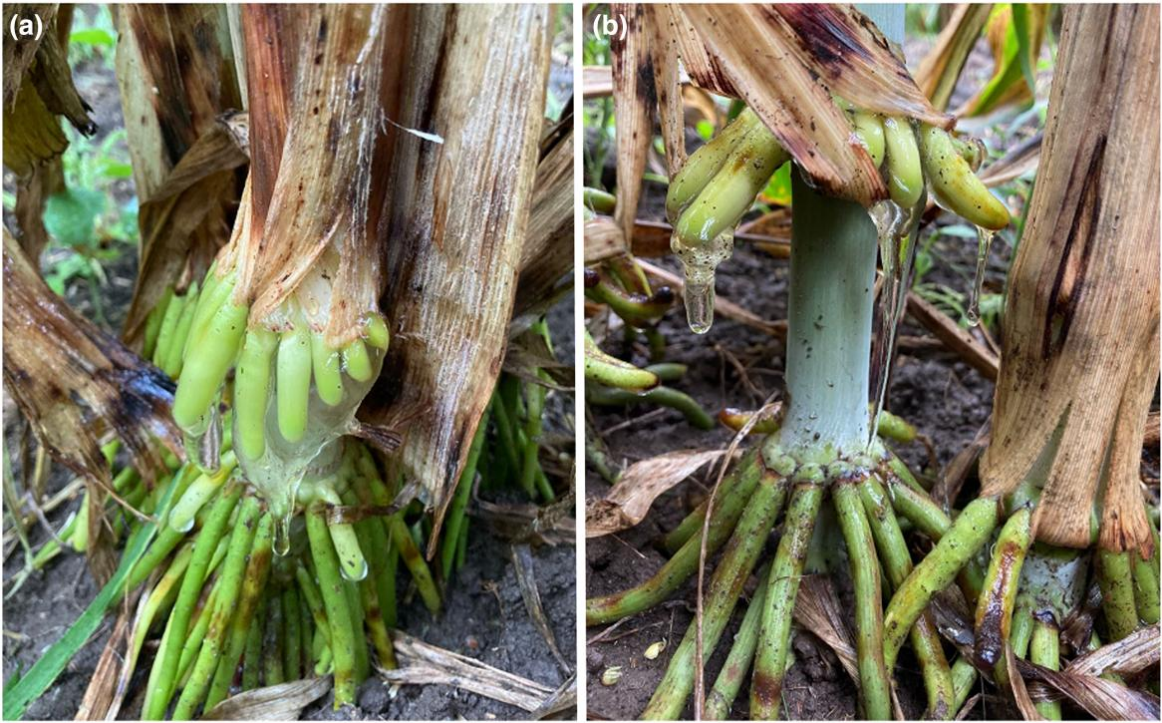
Mucilage production on aerial roots of minicore accessions a) ‘IS27912’ and b) ‘IS14861’ following rain in Madison, Wisconsin.

Sorghum (*Sorghum bicolor* (L.) Moench) is the fifth most cultivated cereal in the world, grown for the production of feed, food, fodder, and the production of renewable fuels and chemicals (Paterson et al. 2009; FAO 2023), often under challenging environmental conditions (heat, drought, low-fertility soils) that are expected to become more prevalent due to climate change (Arora 2019). From past studies of the sorghum minicore, which includes sorghum landraces from 57 different countries (Upadhyaya et al. 2009), we were aware that some accessions could form aerial roots with mucilage, (Fig. 1) and Venado et al. (2023) recently showed they could support diazotrophs like the Sierra Mixe maize. The genetic basis underlying the production of aerial roots is not well-documented, yet it is plausible that the ability to form aerial roots with mucilage is evolutionarily conserved given that maize and sorghum diverged from the most recent common ancestor 12 MYA (Swigoňová et al. 2004),

We have employed a genome-wide association study (GWAS) of 2 panels of genetically diverse sorghum genotypes to identify the genetic basis of aerial root-associated traits that enable sufficient nitrogen fixation as documented in maize. In parallel, we evaluated the environmental factors influencing these aerial root traits in sorghum. Furthermore, we analyzed the monosaccharide composition of mucilage polymers secreted from multiple sorghum landraces as a function of the amount of fertilizer applied to the plots. A better understanding of the genetic basis and environmental effects on these traits will support the long-term goal of enabling BNF in the mucilage of aerial roots to make sorghum production more sustainable.

## Materials and methods

### Field experimental design and phenotype measurements

An initial evaluation of 233 accessions of the sorghum minicore (Upadhyaya et al. 2009) and 406 accessions of the sorghum association panel (SAP) (Casa et al. 2008) at the UF North Florida Research and Education Center-Suwannee Valley (30.3°N, −82.9°W) in the summer of 2021 revealed that the SAP only contained 16 accessions that displayed aerial roots. A subset of 30 SAP accessions—the 16 aerial root-producing accessions and 14 closely related accessions that do not produce aerial roots—were selected for subsequent studies in the summer of 2022 at the same Florida location and the University of Wisconsin West Madison Agricultural Research Station (43.06°N, −89.5°W). The site in Florida has sandy soil whereas the site in Wisconsin has gravelly sandy loam soil (USDA, 2002; USDA, 2006). A split-plot design was used at both locations, with 1/2 of the plot receiving the standard recommended fertilizer application and the other half of the plot 50% of the standard level to mimic a low-input agricultural production system. The standard fertilizer application in Florida comprised 127, 206, and 134 kg ha^−1^ of nitrogen, phosphorus, and potassium, respectively. The standard fertilizer was applied at planting, 40 days post-planting, and 80 days post-planting. In Wisconsin, the standard fertilizer comprised 239, 44, and 44 kg ha^−1^ of nitrogen, phosphorus, and potassium, respectively, applied 30- and 60-days post-planting. At both locations, each main plot was divided into 2 replicates in which the 263 selected sorghum accessions were planted in randomized order. The sweet sorghum cultivar ‘M81E’ (Broadhead et al. 1981) was included as a check and spaced regularly throughout the field to identify gradients in the field that might impact plant growth and development, including the aerial root phenotypes. The plants were cultivated in rows of 1.5 m in length. Plants within a row were thinned to an inter-plant spacing of approximately 10 cm. The inter-row spacing was 76 cm. Supplemental irrigation was provided as needed to prevent drought stress. Plants were phenotyped at the flowering stage, defined as the time when 50% of the plants in the row were producing pollen.

At flowering, 3 central plants in each row were phenotyped for the number of nodes displaying aerial roots, the total number of aerial roots on the upper node, aerial root length (mm), aerial root diameter (mm), and stem diameter (mm) at 1.5 m above the soil level. Two perpendicular measurements were taken for aerial root diameter and stem diameter, and averaged for subsequent analyses. Additionally, aerial root volume was calculated from aerial root length and aerial root diameter with the following formula:


$$V=L\times {\left(\frac{D}{2}\right)}^{2}\times \pi$$

where *V* represents the aerial root volume, *L* is the aerial root length, and *D* is the diameter of the aerial root, calculated as the average of 2 perpendicular measurements.

### Analysis of variance (ANOVA) of factors influencing aerial root traits

An ANOVA was performed to determine the effect of location, fertilizer treatment, and genotype on the number of nodes displaying aerial root, number of aerial roots on the upper node, aerial root diameter, aerial root length, and aerial root volume. Genotypes that did not display aerial roots under any of the conditions were not included in the ANOVA. The following model was used:


$$Y_{ijk}=\mu +\alpha_{i}+\beta_{j}+\gamma_{k}+\varepsilon_{ijk}$$

where *Y_ijk_* denotes the observation in location *i* (Wisconsin or Florida), at fertilizer level *j* (standard or reduced) for genotype *k* (from the 73 accessions that produced aerial roots), *μ* denotes the mean, *α_i_* the location effect, *β_j_* the treatment effect, *γ_k_* the genotype effect, and *ε_ijk_* the residual. Measurements in each replicate were averaged to account for biological variation, and averages were log-transformed to fit a normal distribution. The ANOVA was performed using a mixed model in JMP v. 16 software (SAS Institute Inc., Cary, NC, 1989–2022).

### Association mapping and heritability estimation

Genotyping-by-sequencing (GBS) data were obtained from Hu et al. (2019) for the minicore and SAP accessions. Missing genotype data were imputed with the linkage disequilibrium k-nearest neighbor genotype imputation method (LD-kNNi) (Money et al. 2015) using Trait Analysis by aSSociation, Evolution, and Linkage (TASSEL) v5.2.87 (Bradbury et al. 2007). Following the removal of SNPs with greater than 40% unknown alleles and a minor allele frequency of less than 5%, 101,717 SNPs were utilized in all association analyses. The first 3 principal components (PCs) calculated with TASSEL v5.2.87 were used to construct a population structure matrix (Q) (Pritchard et al. 2000; Endelman and Jannink 2012). TASSEL v5.2.87 was used to generate a kinship matrix (*K*) between accessions using centered identity-by-state. Association analyses were conducted with TASSEL v5.2.87 on the combined multi-environmental data from 2022, following a compressed mixed linear model (CMLM) with P3D variance component estimates based on mean scores of phenotypic values (Yu et al. 2006; Zhang et al. 2010). SNPs with significant associations were determined using the Bonferroni correction (Dunn 1959) and false-discovery-rate-adjusted *P*-values with a threshold of 0.05 (Benjamini and Hochberg 1995). Significant SNPs were associated with candidate genes using the average linkage disequilibrium block size of the minicore (Wang et al. 2013) and the sorghum BTx623 reference genome v3.1.1 (Phytozome v13, Joint Genome Institute) (Supplementary Fig. 10a) (Paterson et al. 2009; McCormick et al. 2018). The expression profiles of candidate genes were evaluated with the European Molecular Biology Laboratory-European Bioinformatics Institute (EMBL-EBI) expression atlas (Moreno et al. 2022; EMBL-EBI 2023). Orthologs of the sorghum candidate genes were identified with OrthoDB v11 (Kuznetsov et al. 2023). Amino acid sequences predicted based on the DNA coding sequences were aligned using the multiple sequence comparison (MUSCLE) algorithms in Molecular Evolutionary Genetics Analysis (MEGA) v11 (Edgar 2004; Tamura et al. 2021). Maximum-likelihood phylogenetic trees were constructed using the aligned amino acid sequences with MEGA11, and tree topology was evaluated with bootstrap sampling analyses with 1,000 replicates (Tamura et al. 2021).

### Statistical analysis for association mapping

The CMLM used to evaluate aerial root traits was:


y=Xα+Kμ+ε

where *y* represents the vector of phenotypic values; *X* represents the genotype; *α* is the vector of fixed effects [genetic marker information and the population structure (Q matrix)]; *K* is the relative kinship matrix; *µ* is the vector of random additive genetic effects, and ε is the experimental residual error.

Broad-sense heritability was calculated using the following formula:


$$H^{2}=\;\frac{\sigma_{G}^{2}}{\sigma_{G}^{2}+\frac{\sigma_{E}^{2}}{L}}$$

where *σ*^2^*_G_* represents the genetic variance; *σ*^2^*_E_* is the residual variance, and *L* is the number of locations (Bernardo 2020).

### Plant material and DNA extractions for aerial root inheritance studies

Initial crosses between the landrace ‘IS23992’ (male) and the sweet sorghums ‘UF15’ and ‘UF20’ (Vermerris et al. 2011; Castro et al. 2017) were performed by applying pollen from the male parent on panicles of the female parent after emasculation, induced by covering the panicle with a plastic bag prior to flowering, as described by Schertz and Clark (1967). The F_1_ progenies and F_2_ populations were cultivated at the UF North Florida Research and Education Center-Suwannee Valley in 3-m rows with 10 cm between plants and 76 cm between rows. Fertilizer (127 kg ha^−1^ of nitrogen, 206 kg ha^−1^ of phosphorus, and 134 kg ha^−1^ of potassium) was applied at planting. Following the initial crosses, the F_1_ progenies were cultivated and self-pollinated to generate the F_2_ populations (Supplementary Fig. 6). F_2_ plants were phenotyped for the presence of aerial roots, and leaf tissue was collected from 100 F_2_ plants, frozen and ground in liquid nitrogen, and stored at −80°C. Genomic DNA was isolated using a GenElute plant genomic DNA miniprep kit (Sigma-Aldrich, St Louis, MO) and stored at −20°C.

### Partial sequencing of GWAS SNPs and promoter analyses

The GBS data from Hu et al. (2019) were used to identify the allelic states of ‘IS23992’ for S3_69358462 and S7_58991587 associated with the number of nodes with aerial roots. Primers were designed with Oligo v7 software (Molecular Biology Insights, Inc., Cascade, CO) to amplify a fragment of 350 bp containing S3_69358462 or S7_58991587 (Supplementary Table 5). PCR amplification was performed on ‘IS23992’, ‘UF15’, and ‘UF20’ with RedAccu*Taq* LA DNA polymerase (Sigma-Aldrich, St Louis, MO) with a Bio-Rad C1000 Touch Thermal Cycler (Bio-Rad Laboratories, Inc., Hercules, CA) with the following protocol: initial denaturation at 98°C for 30 s; 35 cycles of denaturation at 94°C for 15 s, annealing at 62°C for 20 s, and extension at 68°C for 30 s; followed by a final extension of 68°C for 10 minutes and a hold at 4°C. PCR products were visualized on a 2% (w/v) agarose gel with GelRed nucleic acid stain (Sigma-Aldrich, St. Louis, MO). The PCR product was purified with DNA clean & concentration-5 kit (Zymo Research, Irvine, CA) and sequenced (Azenta Life Sciences, South Plainfield, NJ). Sequences were aligned and visualized with SnapGene v6.2.2 (GSL Biotech, LLC, San Diego, CA). Putative *cis*-regulatory elements in the promoter region of *Sobic.003G379700* were evaluated with New PLant *cis*-ACting regulatory DNA Elements (PLACE) v30 using the region 3,000 bp upstream of the transcription start site (Higo et al. 1999).

### SSR markers associated with S7_58991587

Three SSR markers (SSR_5832405, SSR_58891366, and SSR_59038757) developed by Yonemaru et al. (2009) were in proximity to S7_58991587. The GenBank accession number, SSR motif, and primer sequence were described by Yonemaru et al. (2009). Primers were synthesized by Sigma-Aldrich (St. Louis, MO) (Supplementary Table 5). PCR amplification of the genomic DNA of the 100 F_2_ plants was performed with Red*Taq* ReadyMix (Sigma-Aldrich, St Louis, MO) with a Bio-Rad C1000 Touch Thermal Cycler using the following protocol: initial denaturation at 95°C for 1 minute; 35 cycles of 95°C for 10 s, annealing for 20 s, 72°C for 30 s; followed by a final extension at 72°C for 3 minutes and a hold at 4°C. Annealing temperatures were 60°C for SSR_5832405 and SSR_58891366, and 62°C for SSR_59038757. PCR products were visualized on a 4% (w/v) GenePure HiRes agarose gel (BioExpress, Kaysville, UT) with GelRed nucleic acid stain. The allelic states of the 3 SSR loci in the F_2_ plants were compared to those of the parents of the F_2_ populations, ‘IS23992’, ‘UF15’, and ‘UF20’ (Supplementary Fig. 10b). The rate of recombination between SSR markers was calculated with the following formula:


$$\mathrm{Rate}\;\mathrm{of}\;\mathrm{recombination}\;=\;\frac{\mathrm{number}\;\mathrm{of}\;\mathrm{recombinants}}{\mathrm{total}\;\mathrm{number}\;\mathrm{of}\;\mathrm{offspring}}\;\times 100.$$

### χ^2^ goodness-of-fit test for aerial root formation

The observations on the presence of aerial roots in the 2 F_2_ populations, ‘UF15’ × ‘IS23992’ and ‘UF20’ × ‘IS23992’, were used to test whether the presence of aerial roots in these populations was inherited as a single dominant trait. The hypothesized (expected) segregation ratio in the F_2_ populations was 3:1, with 1 representing plants without aerial roots. A χ^2^ goodness-of-fit test (Mather 1951) with 1 d.f. was performed:


$$X^{2}=\;\overset{n}{\underset{1}{\sum }}\frac{{\left(O-E\right)}^{2}}{E}$$

degreesoffreedom=(numberofphenotypes)−1

where *O* is the observed number of F_2_ plants with aerial roots and *E* is the expected number of plants with aerial roots under the null hypothesis.

### Monosaccharide analysis of aerial root mucilage polymers

Water-released aerial root mucilage was collected and dried in pre-weighed 2-mL safe-lock tubes for 10 minicore accessions that produced aerial roots in contrasting fertilizer treatments from the same plots in Florida used for the phenotyping experiments. The alcohol-insoluble residue (AIR), representing polysaccharides as opposed to low-molecular weight carbohydrates, was prepared from the dried mucilage as described by Polko et al. (2018). In brief, samples were ground using a ball mill and washed twice with 70% (v/v) ethanol, once with chloroform:methanol (1:1 v/v), and once with acetone. After drying, total mucilage AIR was weighed and re-suspended in 1.5-mL water. Samples (150-µL aliquots) were mixed with 150 µL of 4-M trifluoroacetic acid (TFA) in a 2-mL screw-cap tube to hydrolyze the matrix polysaccharides for 90 minutes at 120°C (Voiniciuc and Günl 2016). Samples and 9-sugar mixes containing ribose as internal standard were separated via high-performance anion-exchange chromatography with pulsed amperometric detection (HPAEC-PAD) using a Metrohm 940 Professional IC Vario system (Riverview, FL) equipped witha Metrohm Metrosep Carb 2–250/4.0 analytical and guard columns, and a solvent gradient as described by Mielke et al. (2021). The relative composition (mol %) was calculated by factoring in the molecular weight of each monosaccharide (Voiniciuc and Günl 2016). A Student's *t*-test was employed to determine if statistically significant differences existed between the mucilage composition of the 10 accessions or between fertilizer treatments.

## Results

### Diversity in aerial root formation among sorghum landraces and breeding lines

To prepare for the GWAS in 2 geographic locations and with a standard and reduced level of fertilizer, 2 panels of genetically diverse sorghums were subjected to an initial phenotypic evaluation in Florida in 2021: the SAP, consisting of 406 modern sorghums and US breeding lines (Casa et al. 2008) and the sorghum minicore, a collection of 242 landraces (Upadhyaya et al. 2009). This initial screening identified 16 aerial root-producing accessions in the SAP compared to 137 accessions in the minicore (Supplementary Fig. 1^1^).

Given the low proportion of accessions with aerial roots in the SAP, a subset of 30 genotypes were selected for a replicated phenotypic screening in the summer of 2022: the 16 accessions identified in 2021 and 14 closely related accessions that did not form aerial roots. These 30 accessions together with 233 accessions from the minicore (9 original accessions did not generate enough seed) were phenotyped in Florida and Wisconsin (Supplementary Table 1).

### Environmental factors influence aerial root traits in sorghum

**Fig. 2.**
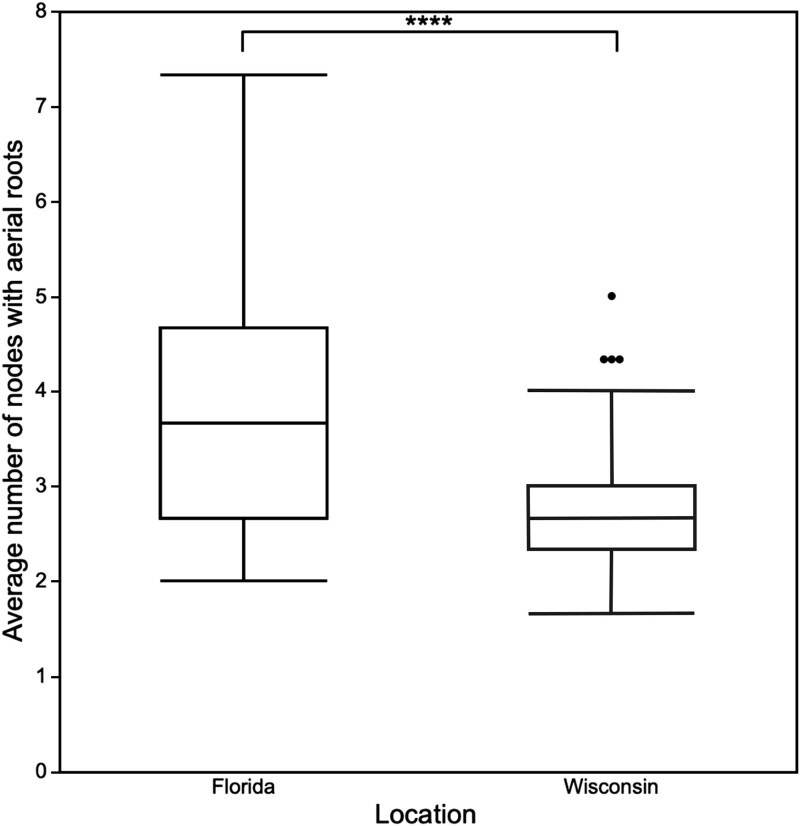
Box plot of the average number of nodes with aerial roots in Wisconsin and Florida. The box represents the interquartile range, the line within the interquartile range represents the median number of nodes, the whiskers of the box plot indicate the range of the number of nodes, and the outside dots indicate outliers of the dataset. The 4 asterisks represent a statistically significant difference between locations based on a 2-tailed Student's *t*-test (*P* ≤ 0.0001).

The fact that 16 accessions from the SAP displayed aerial roots in Florida in 2021, but only 3 were identified as producing aerial roots consistently in both Florida and Wisconsin in 2022, combined with the small number of accessions that produced aerial roots in both Wisconsin and Florida (Supplementary Table 1) provided evidence for an environmental effect on aerial root formation. An ANOVA was conducted to determine the effect of genotype, location and treatment on the aerial root-related traits. The results of the ANOVA are summarized in Table 1. The ANOVA of the number of nodes with aerial roots revealed that both genotype and location have a significant effect on this trait (Table 1). A comparison of the genotypes that produced statistically significantly more or fewer nodes with aerial roots than the overall mean indicated that they belonged to the durra and caudatum races. However, there was no association between the individual race and the number of nodes with aerial roots. In other words, race was not predictive of the phenotype. There was also no apparent association with the geographic origin of these accessions (Table 2). Significant differences observed between the 2 locations indicated that, on average, the plants produced more nodes with aerial roots in Florida than in Wisconsin (Fig. 2). This result demonstrates that environmental differences (including soil type, soil microbiome, temperature, precipitation, humidity, day length, wind speed) can influence the number of nodes producing aerial roots.

**Table 1.** ANOVA of aerial root traits by location, fertilizer treatment, and genotype.

Sources	d.f.	Number of nodes with aerial roots	Number of aerial roots on the upper node	Aerial root length	Aerial root diameter	Aerial root volume
Genotype	72	*0.041*	0.972	0.138	*0*.*0002*	0.353
Location	1	*0*.*0002*	0.378	0.114	*<0*.*0001*	*0*.*0004*
Treatment	1	0.283	0.232	0.194	0.824	0.464

The number of genotypes was the subset of the population that made aerial roots in at least 1 of the locations or fertilizer treatments. Statistically significant effects on traits (*P* ≤ 0.05) are marked in italics.

**Table 2.** The average number of nodes forming aerial roots on selected sorghum accessions cultivated in Florida and Wisconsin, under standard or reduced fertilizer levels.

Accession	Florida		Wisconsin		Origin	Race
	Standard fertilizer	Reduced fertilizer	Standard fertilizer	Reduced fertilizer		
IS2382	2.0	0	0	0	South Africa	Caudatum
IS4631	2.0	0	0	0	India	Durra
IS5094	2.0	0	0	0	India	Durra
IS5386	2.5	0	0	0	India	Durra
IS15170	6.3	0	0	0	Cameroon	Caudatum
IS22609	2.0	0	0	0	Sri Lanka	Caudatum
IS23891	0	0	0	4.3	Yemen	Durra
IS25732	7.3	0	0	0	Mali	Durra
IS29091	0	0	4.2	3.7	Yemen	Durra-caudatum
IS31706	0	0	4.2	4.3	Yemen	Durra
*IS23992*	*2*.6*	*2.0*	*3.0*	*1.0*	*Yemen*	*Caudatum*
*IS20679*	*0*	3.2*	*0*	*0*	*USA*	*Guinea-caudatum*
*IS27034*	*0*	*0*	*0*	*2.3*	*Sudan*	*Durra*

The first 10 genotypes were identified in the ANOVA as having significantly different numbers of nodes with aerial roots compared to the mean. The genotypes in italics were selected as parents for breeding populations. The origin and race of the accessions are based on Upadhyaya et al. (2009).

*Based on 2021 data.

**Fig. 3.**
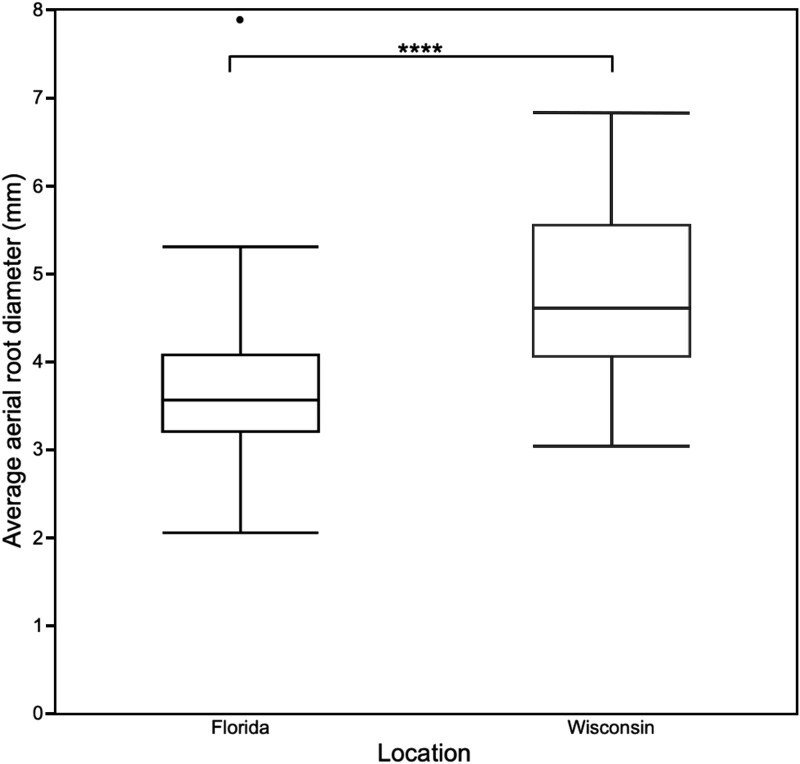
Box plot of average aerial root diameter in Wisconsin and Florida. The box represents the interquartile range, the line within the interquartile range represents the median aerial root diameter, the whiskers indicate the range of aerial root diameter, and the outside dot indicates an outlier of the dataset. The 4 asterisks represent statistically significant differences between locations based on a 2-tailed Student's *t*-test (*P* ≤ 0.0001).

**Fig. 4.**
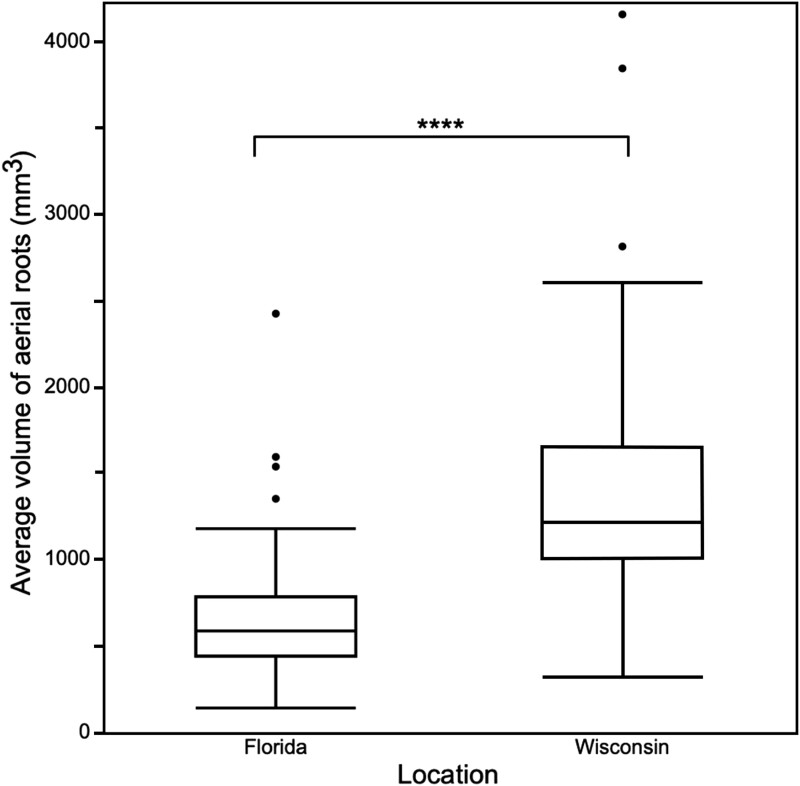
Box plot of average aerial root volume in Wisconsin and Florida. The box represents the interquartile range, the line within the interquartile range represents the median aerial root volume, the whiskers indicate the range of aerial root volume, and the outside dots indicate outliers of the dataset. The 4 asterisks represent statistically significant differences between locations based on a 2-tailed Student's *t*-test (*P* ≤ 0.0001).

Furthermore, the ANOVA indicated genotype significantly impacts aerial root diameter. The genotypes with aerial roots whose diameters were statistically significantly greater or smaller than the overall mean belonged to several different races (durra, caudatum, bicolor, guinea) and represented multiple geographic origins (Table 2). Location also affected aerial root diameter significantly, with, on average, aerial roots in Wisconsin displaying a greater diameter than in Florida (Fig. 3). Consequently, the location had a statistically significant effect on aerial root volume (a function of the diameter), with a greater average volume in Wisconsin (Fig. 4).

The plants cultivated under reduced fertilizer levels were overall paler green than the plants that had received the standard level of fertilizer. The fertilizer level, however, did not have a significant impact on aerial root traits when considering the entire collection of genotypes (Table 1). Nonetheless, it is apparent from Table 2 that individual genotypes responded differently to the amount of fertilizer that was applied. Given the desire to use BNF as a mechanism to reduce the use of synthetic fertilizer, sorghum genotypes producing aerial roots under reduced nitrogen conditions are the most relevant, and they were the basis for the GWAS.

### Genome-wide association analyses of aerial root traits in sorghum

Association mapping of aerial root traits using 101,717 SNPs and a CMLM accounting for population structure (Q) and kinship (*K*) to minimize spurious associations and maximize power, detected marker-trait associations for the number of nodes with aerial roots (nodes) and aerial root diameter under reduced fertilizer conditions. PCA was employed to adjust for differences in allele frequencies across different ancestral populations (races), explaining 8.4 and 5.0% of the genetic variance with the first 2 principal components (Supplementary Fig. 1). The quantile–quantile (Q–Q) plots indicate the model adequately accounted for population structure and kinship, as the observed −log_10_(*P*-value) largely matched the expected −log_10_(*P*-value) (Supplementary Fig. 2). The SNPs with significant associations, allele frequencies, and the portion of phenotypic variance explained by the SNPs for each phenotype are listed in Supplementary Table 2. The phenotypic variance explained by S3_69358462 and S7_58991587 associated with the number of nodes with aerial roots under reduced nitrogen conditions was 19 and 30%, respectively. Furthermore, S3_1871493 and S3_11242543 explain 17 and 20% of the phenotypic variance for aerial root diameter. SNPs associated with the various aerial root phenotypes were located within or proximal to candidate genes with molecular functions related to root system architecture, phytohormones, transcriptional regulation, and stress tolerance. The SNP-based broad-sense heritability (H^2^) was 0.69 and 0.70 for the number of nodes that form aerial roots and aerial root diameter, respectively. Supplementary Table 3 lists the genotypes utilized in the GWAS, the SNP allele for those genotypes at the loci displaying a statistically significant association with the phenotype, the average number of nodes with aerial roots and the average aerial root diameter for each genotype. The relationship between the allelic states and the phenotypes is summarized in Supplementary Fig. 3.

### Evaluating the inheritance of aerial roots in 2 F_2_ populations

**Fig. 5.**
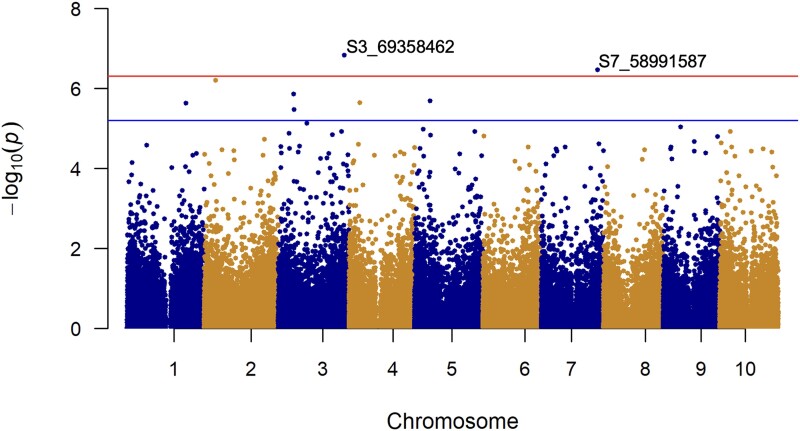
Genome-wide association study for the number of nodes with aerial roots under reduced fertilizer conditions. Each dot represents a SNP. The Manhattan plot displays 2 SNPs above the Bonferroni-corrected threshold (red line). The blue line represents the significance threshold for the false discovery rate.

Two F_2_ populations, ‘UF15’ × ‘IS23992’ and ‘UF20’ × ‘IS23992’, were used to evaluate the marker-trait relationship of the SNPs S3_69358462 and S7_58991587 with the number of nodes forming aerial roots (Fig. 5). The sweet sorghums ‘UF15’ and ‘UF20’ do not produce aerial roots, whereas the landrace ‘IS23992’ consistently produces multiple nodes with aerial roots across environments (Wisconsin vs Florida and reduced vs standard fertilizer treatments). The ratios of the number of F_2_ plants with aerial roots to the number of F_2_ plants without aerial roots in these 2 populations were 84:34 and 151:39, respectively. A χ^2^ goodness-of-fit test indicated that these observations were consistent with the null hypothesis of a Mendelian segregation ratio of 3:1 (aerial roots:no aerial roots), implying that aerial root formation is inherited in these populations as a single dominant trait (Table 4).

**Table 3.** The average aerial root diameter (in mm) of sorghum accessions cultivated in Florida and Wisconsin, under standard or reduced fertilizer levels.

Accession	Florida		Wisconsin		Origin	Race
	Standard fertilizer	Reduced fertilizer	Standard fertilizer	Reduced fertilizer		
IS1219	–	–	3.7	–	China	Guinea-bicolor
IS4060	–	–	–	3.0	India	Durra-bicolor
IS5094	2.5	–	–	–	India	Durra
IS5667	–	–	4. 5	4.1	India	Durra
IS5919	5.0	–	–	–	India	Durra
IS12804	2.1	2.1	4.2	3.7	Turkey	Bicolor
IS14861	4.2	4.8	6.0	–	Cameroon	Caudatum
IS26025	6.0	–	–	–	Mali	Guinea
IS27697	–	–	–	6.8	Sierra Leone	Guinea
IS28747	–	–	–	3.2	Yemen	Durra-caudatum
IS30417	–	–	3.3	3.3	China	Caudatum-bicolor
IS30443	3.1	–	3.6	–	China	Caudatum-bicolor
IS30451	2.8	–	–	4.0	China	Caudatum-bicolor
PI276837	–	5.3	–	–	Ethiopia	Durra-caudatum

This list of genotypes was identified in the ANOVA as having significantly different aerial root diameters compared to the mean. Diameter measurements were averaged between replicates. The origin and race of the accessions are based on Upadhyaya et al. (2009). Absence of aerial roots is denoted with a dash.

**Table 4.** χ^2^ goodness-of-fit test for a 3:1 phenotypic ratio for aerial root emergence in 2 F_2_ populations.

F_2_ population	Population size	Observed plants with aerial roots	Expected plants with aerial roots	$\chi^{2}$	*P*-value
UF15 × IS23992	118	84	88.5	0.915	0.339
UF20 × IS23992	190	151	142.5	2.028	0.154

**Fig. 6.**
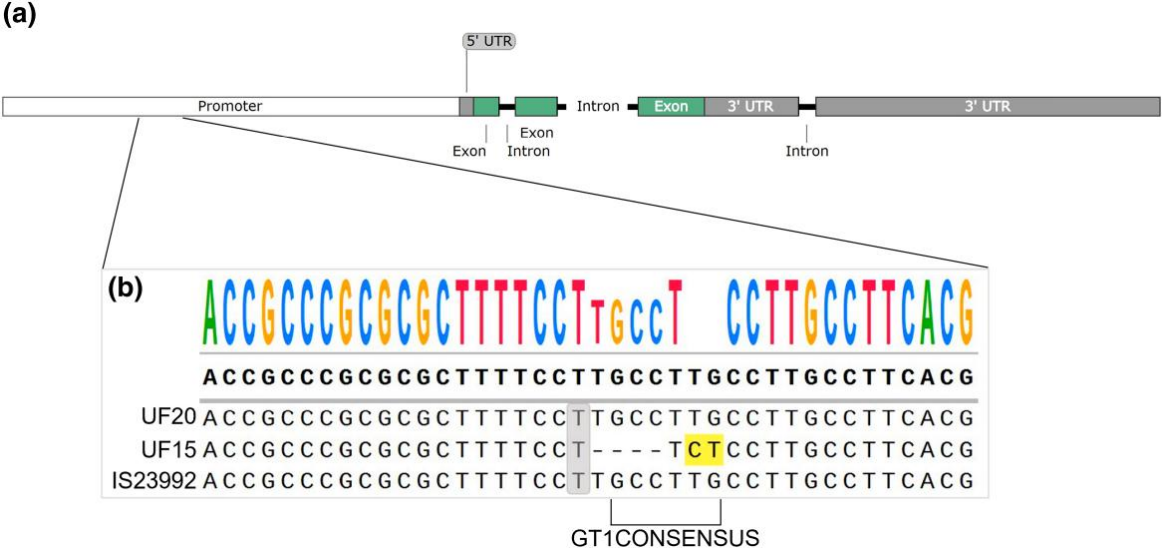
Schematic of the location of S3_69358462 in the promoter region of *Sobic.003G379700*. a) Annotated genomic sequence of *Sobic.003G379700* visualized with SnapGene. The promoter is displayed in white, the 5′ and 3′ UTR in gray, exons in green, and introns in black. b) Sequence alignment of the region around S3_69358462 located in the promoter of *Sobic.003G379700*. The consensus sequence is displayed at the top of the alignment, where each nucleotide is represented by a different color and letter size reflects the number sequences on which the consensus is based. S3_69358462 is highlighted in gray. Dashes represent deletions, whereas nucleotides highlighted in yellow do not match the consensus sequence. Below the alignment, the region of the *GT1CONSENSUS cis*-regulatory element is outlined.

Based on the GBS data, ‘IS23992’ has the reference SNP allele for S3_69358462, which is predicted to have zero effect on the number of aerial roots. Sequencing across S3_69358462 confirmed that the allele for ‘IS23992’ matched the GBS data and revealed that ‘UF15’ and ‘UF20’ possess the same allele at this locus. SNP S3_69358462 is located 2,325 bp upstream of the transcription start site of *Sobic.003G379700*. The promoter of this gene was evaluated for *cis*-regulatory elements using New PLACE v30, which identified the *GT1CONSENSUS* (5′-GRWAAW-3′) element 2 bp downstream of S3_69358462. Sequencing analyses identified that ‘UF15’ has a 4-bp deletion inside the *GT1CONSENSUS cis*-regulatory element in the promoter of *Sobic.003G379700* (Fig. 6).

The absence of polymorphisms at SNP locus S3_69358462 and the presence of the reference allele in ‘IS23922’ led us to 2 hypotheses: In these F_2_ populations, the presence of aerial roots is either controlled solely by the locus on chromosome 7, or aerial root formation is a complex trait where S3_69358462 contributes less to the phenotype than S7_58991587.

**Fig. 7.**
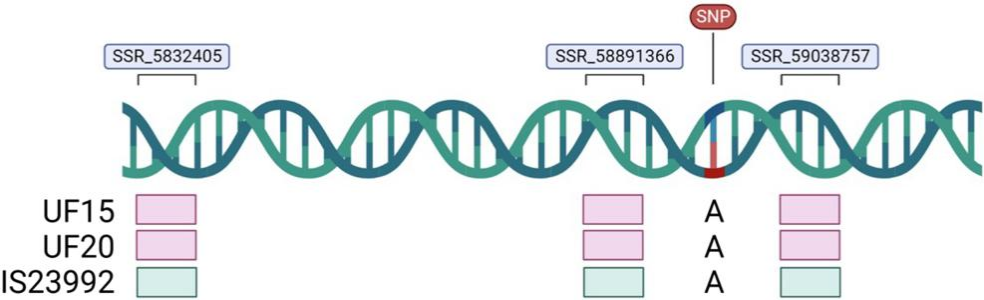
Schematic indicating the relative location of S7_58991587 and the SSR markers deployed to evaluate the inheritance of aerial roots. The numbers represent the genome coordinates in bp; the figure is not drawn to scale. Sequencing results for the accessions ‘UF15’, ‘UF20’, and ‘IS23992’ are shown below the location of S7_58991587. The different SSR alleles are represented by boxes of different colors displayed below their respective SSR marker. This figure was created with BioRender.

Sequencing of SNP S7_58991587 in ‘IS23992’ to evaluate the inheritance of this locus in the 2 F_2_ populations revealed the presence of the A allele, which is also present in ‘UF15’ and ‘UF20’. This result, however, contradicted the GBS data (Hu et al. 2019) for ‘IS23992’, which indicated the T allele at this position. Since genotypes with aerial roots have the T allele (Supplementary Fig. 3), we relied on SSR markers in proximity to S7_58991587 to evaluate the F_2_ populations. Three SSR markers on chromosome 7 (SSR_5832405, SSR_58891366, and SSR_59038757) were identified to be polymorphic (‘IS23922’ vs ‘UF15’ and ‘UF20’), co-dominant, reproducible, and locus-specific (Fig. 7). An evaluation of 100 F_2_ individuals from the 2 F_2_ populations (‘UF15’ × ‘IS23992’ and ‘UF20’ × ‘IS23992’) indicated that 56 of the 61 individual plants that produced aerial roots harbored at least 1 ‘IS23992’ SSR allele at each of the 3 SSR loci (i.e. homozygous for the ‘IS23992’ SSR allele or heterozygous), with no recombination between the SSR markers (Supplementary Table 4). Of the remaining plants that produced aerial roots, a 5% recombination rate was observed between SSR_5832405 and SSR_58891366, while no recombination events were detected between SSR_58891366 and SSR_59038757. Furthermore, no double recombination events were observed for the 61 F_2_ plants that produced aerial roots. Of the 100 F_2_ individuals, 39 made brace roots but not aerial roots, with 24 harboring the ‘UF15’ and ‘UF20’ SSR alleles for all 3 SSR markers. Eight of the 39 F_2_ plants producing brace roots but not aerial roots harbored the ‘IS23992’ SSR allele at each SSR marker rather than the ‘UF15’ or ‘UF20’ SSR allele. Recombination rates of 3 and 1% were observed between SSR_5832405 and SSR_58891366 and between SSR_58891366 and SSR_59038757, respectively, for plants phenotyped to produce brace roots, with 1 double recombinant. The SSRs evaluated on chromosome 7 segregated with the aerial root phenotype 97%, 87%, and 93% of the time, for the SSRs, SSR_5832405, SSR_58891366, and SSR_59038757, respectively (Supplementary Fig. 4).

### Monosaccharide composition of sorghum aerial root mucilage

To investigate if the composition of aerial root mucilage varied depending on the fertilizer level, the AIR of secreted polymers was isolated for 10 accessions of the minicore that produced aerial roots under both standard and reduced fertilizer conditions in Florida. The mucilage polymers consisted primarily of galactose, arabinose, and fucose, with lower proportions of mannose, xylose, glucose and glucuronic acid (Table 5; Supplementary Table 6). The results suggest that sorghum aerial root mucilage is rich in arabinogalactans that are likely fucosylated, or that it is a mixture of several polysaccharides. The monosaccharide composition of the mucilage polymers showed minimal variation across the 10 accessions and the 2 fertilizer treatments (Table 5).

**Table 5.** Average relative composition with standard deviations of monosaccharides that are present in the aerial root mucilage of sorghum landraces cultivated at 2 fertilizer levels.

	Relative composition (mol %)						
	Fuc	Gal	Ara	Glc	Xyl	Man	GlcA
Standard fertilizer	17 ± 1	37 ± 4	23 ± 1	5 ± 2	6 ± 2	6 ± 1	6 ± 1
Reduced fertilizer	16 ± 2	38 ± 4	24 ± 2	5 ± 2	5 ± 2	6 ± 1	6 ± 1

Complete data are in Supplementary Table 6.

Fuc, fucose; Gal, galactose; Ara, arabinose; Glc, glucose; Xyl, xylose; Man, mannose; GlcA, glucuronic acid.

## Discussion

### Location and genotype significantly impact aerial root traits

We conducted a statistical analysis of 2 diverse sorghum collections in Florida and Wisconsin cultivated under standard and reduced fertilizer management strategies to determine the effect of genotype, location, and fertilizer treatment on the aerial root-related traits. Our results indicate that genotype and location have a significant impact on critical traits that have been shown to influence the efficacy of BNF in maize, such as the number of nodes producing aerial roots and aerial root diameter (Table 1) (Pankievicz et al. 2022). In maize, it has been demonstrated that specific landraces can produce more nodes with aerial roots compared to modern lines (Pankievicz et al. 2022), which we also observed in the SAP relative to the minicore (Supplementary Fig. 1^1^). These observations underscore the potential of landraces for allele mining (Cuevas et al. 2024). Since both collections of sorghum represent the different races of sorghum (bicolor, caudatum, durra, guinea, kaffir), the low number of accessions forming aerial roots in the SAP could imply that the ability to develop aerial roots has been under negative selection in modern breeding programs from which the accessions in the SAP were obtained. However, a comparison of the frequencies of the alleles associated with the aerial root formation in the 2 populations (Supplementary Table 3) did not provide evidence in support of a selective sweep. At this time, we cannot exclude the possibility that aerial root formation has negative effects on agronomic performance (yield, maturity), but additional studies will be required to examine this.

Even though aerial roots were observed among all sorghum races, the most significant variation in the number of aerial roots amongst landraces was observed in accessions belonging to the durra and caudatum races (Table 2). These races originated in the eastern part of Africa, which may represent the origin of the trait or the area where the trait was most beneficial so that genetic diversity was maintained (Morris et al. 2013; Fuller and Stevens 2018; Burgarella et al. 2021). The variation in the diameter of aerial roots among sorghum landraces was not associated with race (Table 3). This makes sense, given that aerial root diameter is contingent on the presence of aerial roots, which is not race-specific.

A significantly higher number of nodes with aerial roots was observed in Florida compared to Wisconsin (Fig. 2). Environmental factors contributing to phenotypic differences amongst locations include soil type, nutrient availability, the soil microbiome, temperature, day length, rainfall, and humidity. In particular, high humidity has been shown to stimulate aerial root formation (Venado et al. 2023). During the summer months, the average relative humidity in Live Oak, Florida ranges from 80 to 85%, compared to 65 to 77% at Madison, Wisconsin.

### GWAS indicate aerial root traits colocalize with loci associated with phytohormones

Considering the significance of genotype in the ANOVA on the number of nodes with aerial roots and aerial root diameter, we performed association mapping of these traits to elucidate their genetic architecture in sorghum. A GWAS following a CMLM detected 2 loci on chromosomes 3 (S3_69358462) and 7 (S7_58991587) associated with the number of nodes with aerial roots under reduced fertilizer conditions (Fig. 5). The SNP-based broad-sense heritability was 0.69, suggesting a substantial portion of the phenotypic variance can be attributed to genetic variance, with estimated SNP effects of 2.58 and 1.81 for S3_69358462 and S7_58991587, respectively (Supplementary Table 2).

SNP S3_69358462 on chromosome 3 resides in the promoter region of candidate gene *Sobic.003G379700*, annotated to encode the transcription factor NAC54 [no apical meristem (NAM), *Arabidopsis thaliana* activating factor 1–2 (ATAF1-2), and cup-shaped cotyledon2 (CUC2)] (Souer et al. 1996; Aida et al. 1997). Analyses of the promoter region of *Sobic.003G379700* indicated that S3_69358462 is 2 bp upstream of a *GT1CONSENSUS cis*-regulatory element (Fig. 6). That element is recognized by GT-1 proteins that have tri-helix DNA-binding domains and that have been shown to induce the expression of genes as a result of environmental stresses in various plant species (Villain et al. 1996; Le Gourrierec et al. 1999; Simpson et al. 2003).

NAC transcription factors regulate a variety of developmental processes as well as biotic and abiotic stress responses (Xiong et al. 2005). *SbNAC54* is the predicted ortholog of *AtNAC032* and *OsNAC48*, and the amino acid sequence of SbNAC54 shares 75 and 83% similarity with AtNAC032 and OsNAC48, respectively (Supplementary Fig. 9). AtNAC032 and OsNAC48 belong to the NAC transcription factor protein family Group I, subfamily ATAF (Ooka et al. 2003). OsNAC48 and AtNAC032 have been characterized to induce root elongation and root system architectural changes via auxin-mediated responses during abiotic stresses (Duval et al. 2002; Guo and Gan 2006; Chung et al. 2009). Auxin acts as a master regulator of root development, as it modulates signaling cascades impacting primary root, lateral root, root hair, and adventitious root morphogenesis (Casimiro et al. 2003; Sibout 2007). *AtNAC032* expression patterns are upregulated under low phosphorus conditions (Fukushima et al. 2017).

In maize the expression of genes encoding NAC transcription factors have been linked to nodal root number and have been identified to be differentially expressed in aerial roots (Zhang et al. 2018; Pankievicz et al. 2022). Following water exposure, the differentially expressed genes *ZmNAC21* and *ZmNAC22*, which are not orthologs of *AtNAC032* and *OsNAC48* but do encode members of the same family of NAC transcription factors, were associated with aerial root mucilage production (Supplementary Fig. 9) (Pankievicz et al. 2022).

Based on transcriptomics data from the accession BTx623, which does not generate aerial roots, *Sobic.003G379700* is highly expressed in a variety of tissues, including the flower and embryo, as well as the roots and shoots during the early stages of development (Supplementary Fig. 5) (Olson et al. 2014).

Sanger sequencing over SNP S3_69358462 in the 2 F_2_ populations, ‘UF15’ × ‘IS23992’ and ‘UF20’ × ‘IS23992’ confirmed the presence of the reference S3_69358462 allele in each genotype (Fig. 6). Given the effect of the reference SNP is zero, we propose that multiple loci control the phenotype of the number of nodes that form aerial roots and that this locus is required for aerial root formation in combination with other loci. Additionally, it is possible that S3_69358462 is not the causal SNP and the fact that the GWAS resulted in an association with this region of the genome could have been due to linkage disequilibrium between the true causal polymorphism and S3_69358462. Since all 3 parents of the F_2_ populations harbor the reference SNP of S3_69358462, this locus is expected to be fixed in the F_2_ populations. Furthermore, the sequencing of the promoter region containing S3_69358462 indicated that ‘UF15’ lacks the *GT1CONSENSUS cis*-regulatory element, which may impact the regulation of *Sobic.003G379700* under reduced nitrogen conditions that may contribute to ‘UF15’ not having the ability to produce aerial roots (Fig. 6b).

On chromosome 7, SNP S7_58991587, associated with the number of nodes with aerial roots, resides in the first exon of gene *Sobic.007G155900*, encoding a basic leucine zipper (bZIP) transcription factor. S7_58991587 is located within a QTL for brace root formation in sorghum (Li et al. 2014). Given the morphological similarities between brace roots and aerial roots, *Sobic.007G155900* may control the development of both types of adventitious roots. *Sobic.007G155900* is the ortholog of Arabidopsis *bZIP36* [*ABSCISIC ACID-INSENSITIVE 5* (*ABI5*)] and *TRAB1* in rice. The amino acid sequence of Sobic.007G155900 shares 48 and 80% similarity with AtbZIP36 and OsTRAB1 (Supplementary Fig. 9). *OsTRAB1* expression is upregulated due to stress, resulting in a protein that modulates abscisic acid (ABA)-induced transcription by binding to ABA response elements (Hobo et al. 1999; Kagaya et al. 2002). OsTRAB1 and AtABI5 are functionally conserved (Finkelstein and Lynch 2000; Lopez-Molina and Chua 2000; Hossain et al. 2010). ABI5 stimulates ABA signaling in Arabidopsis, regulating growth, development, and responses to biotic and abiotic stress (Chen et al. 2020). Under low nitrate conditions in the soil, ABA indirectly regulates lateral root growth in Arabidopsis, where signal transduction is modulated by ABI5 (Signora et al. 2001). Furthermore, ABI5 regulates phosphate absorption through ABA signaling, making it plausible that its expression is upregulated under reduced fertilizer conditions (Zhang et al. 2022). Moreover, nitric oxide negatively regulates ABA signaling to promote root growth via the scavenging of reactive oxygen species (ROS) by the *S*-nitrosylation of AtABI5 and OsTRAB1 (Albertos et al. 2015; Xu et al. 2018).

The detection of S7_58991587 associated with candidate gene *Sobic.007G155900* is consistent with reports that have identified genes that control ripening via ABA signaling as upregulated in maize aerial roots in response to water exposure, indicating aerial root formation and mucilage production in sorghum may be influenced by ABA signaling (Pankievicz et al. 2022). *Sobic.007G155900* in inbred line BTx623, which does not form aerial roots, is highly expressed in all sorghum tissues, with its lowest expression observed in shoot tissue (Supplementary Fig. 5) (Olson et al. 2014). It is important to note that in the transcriptome analyses with BTx623, shoot and root tissue was collected at approximately 8 days post-germination.

Sequencing analyses across S7_58991587 in the parents (‘IS23992’, ‘UF15’, and ‘UF20’) of our F_2_ populations revealed that each genotype harbored the A allele (Fig. 7). These results contradict the GBS data by Hu et al. (2019), who reported that ‘IS23992’ carries the T allele that is associated with the aerial root phenotype. In addition to sequencing errors, this discrepancy could mean that S7_58991587 is not the causal SNP, but simply linked to the causal SNP, or that ‘IS23992’ has a different sequence variant associated with this trait.

Due to the discrepancies between the GBS data and our sequencing data and the possibility that regions flanking S7_58991587 may impact the phenotype, plants from 2 F_2_ populations, ‘UF15’ × ‘IS23992’ and ‘UF20’ × ‘IS23992’ were genotyped with SSR markers near S7_58991587. The co-segregation between the SSR marker genotype and the aerial root phenotype supported the results from the GWAS (Supplementary Table 4). The 8 F_2_ plants that were phenotyped as producing only brace roots, despite harboring the ‘IS23992’ SSR allele for all 3 markers, were either phenotyped incorrectly due to conservative phenotyping or reflect incomplete penetrance, whereby the presence of the alleles associated with aerial root formation is not guaranteed to result in aerial roots under certain environmental conditions. For example, high plant density has been observed to impact aerial root formation negatively. Nonetheless, the identification of these SSR markers associated with the aerial root phenotype could be leveraged in marker-assisted selection, aiding in the selection of sorghums that can produce aerial roots and, therefore, support BNF.

**Fig. 8.**
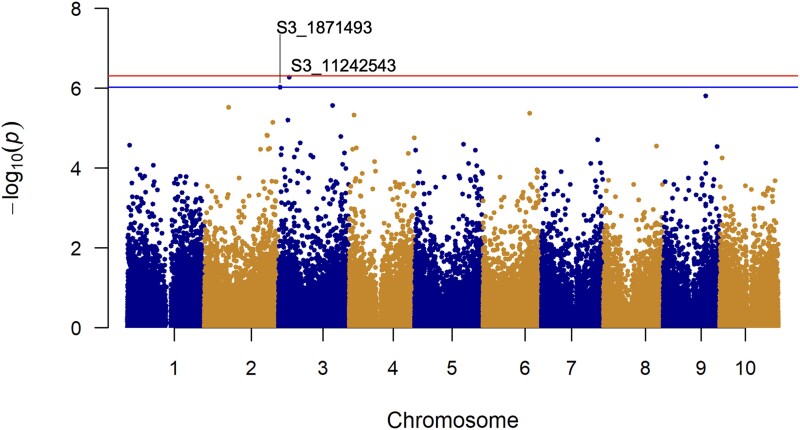
Manhattan plot of GWAS results identifying 2 SNPs that display statistically significant associations with aerial root diameter. Each SNP is represented by a dot. The red and blue lines represent the significance thresholds following Bonferroni correction and fase discovery rate, respectively.

In addition to aerial root formation, aerial root diameter is positively correlated with the ability to produce increased volumes of mucilage that harbors diazotrophs in both maize and sorghum (Pankievicz et al. 2022; Venado et al. 2023). The GWAS evaluating aerial root diameter detected 2 SNPs on chromosome 3, S3_1871493 and S3_11242543 (Fig. 8). S3_1871493 is 21 bp downstream of the candidate gene *Sobic.003G021800*, encoding a bZIP transcription factor, and its amino acid sequence shares 53% similarity to AtbZIP61 (Supplementary Fig. 9). bZIP transcription factors modulate responses to developmental, environmental, and stress signaling (Dröge-Laser et al. 2018). In Arabidopsis, bZIP61 forms a heterodimer with bZIP34, and together this complex can create a network with additional bZIP transcription factors to regulate plant development (Shen et al. 2007; Tong et al. 2021). Specifically, this heterodimer in Arabidopsis can form a network with bZIP29, modulating root meristem activity and root cell number via cell wall organization which may influence aerial root diameter (Van Leene et al. 2016). In BTx623, which does not produce aerial roots, *Sobic.003G021800* is expressed at moderate levels in shoot tissue and low levels in root tissue at 8 days post-germination (Supplementary Fig. 7) (Olson et al. 2014).

SNP S3_11242543 resides 5519 bp upstream of the candidate gene *Sobic.003G123800*, predicted to encode a DE-ETIOLATED-1 (DET1) and DNA damage-binding protein 1 (DDB1)-associated protein 1. Together, DET1 and DDB1 facilitate the degradation of cell differentiation and proliferation regulators in Arabidopsis by acting as components of an E3 ligase. DET1 and DDB1-associated protein 1 is predicted to be a component of the E3 ligase with DET1 and DDB1. In Arabidopsis, the DET1-DDB1 complex negatively affects the stability of ABI5, regulating ABA signaling and impacting root system architecture (Seo et al. 2014). AtDET1 has also been reported to function as a repressor of photomorphogenesis, delaying flowering time (Pepper et al. 1994; Kang et al. 2015). In maize, flowering time is correlated with nodal root emergence (Zhang et al. 2018). Therefore, the identification of candidate gene *Sobic.003G123800* suggests that the timing of the transition from the vegetative to the reproductive stage impacts aerial root formation and aerial root diameter in sorghum. *Sobic.003G123800* expression in BTx623 is restricted to root tissue, where it is expressed at low levels at 3 days post-germination (Turco et al. 2017; Supplementary Fig. 8).

### Sorghum aerial root mucilage composition is stable across genotypes and fertilizer treatments

Venado et al. (2023) demonstrated that sorghum aerial root mucilage is necessary to support BNF. Analysis of monosaccharide composition indicated minimal differences between genotypes and fertilizer treatments (Table 5; Supplementary Table 6). This suggests mucilage composition is kept within a narrow range to afford the properties necessary to support diazotrophic microbes, and this is more easily accomplished if the mucilage is comprised of a single, complex polysaccharide, as opposed to a mixture of polysaccharides with a less complex monosaccharide composition. Our data on sorghum mucilage composition differ from those reported by Xu et al. (2023), who analyzed the mucilage of 2 undisclosed sorghum accessions. Their analysis was, however, based on the composition of soluble carbohydrates in the mucilage rather than on the composition of mucilage polysaccharides.

A comparison between the monosaccharide composition of our sorghum mucilage samples and the reported composition of Sierra Mixe maize aerial root mucilage indicates the 2 sources of mucilage are composed of essentially the same monosaccharides (Van Deynze et al. 2018; Amicucci et al. 2019), but that there are substantial differences in the proportions of individual monosaccharides. Specifically, fucose is the most abundant monosaccharide in maize mucilage (41%), whereas, in sorghum, it is galactose (38 ± 4%) and arabinose (24 ± 2%) (Table 5). Furthermore, glucose is present in sorghum mucilage (5 ± 2%), but was not reported for maize mucilage, possibly because it had not been quantified (Amicucci et al. 2019). Given that the mucilage composition of Sierra Mixe maize appears to be consistent between locations (Van Deynze et al. 2018; Amicucci et al. 2019), the differences in mucilage composition between maize and sorghum are likely species-specific rather than due to the location where the plants were cultivated.

The difference in mucilage composition between maize and sorghum and the observations that the genes identified in gene expression studies of maize aerial roots (Pankievicz et al. 2022) are not the orthologs of the sorghum candidate genes identified in the GWAS (Supplementary Fig. 9) could mean that the ability to support BNF on aerial roots evolved independently in these 2 species.

## Conclusions

Exploring the natural diversity of sorghum for aerial root-related traits that promote BNF is a first step toward reducing agriculture's dependence on synthetic fertilizers and enhancing sorghum as a low-input crop. We observed that the number of genotypes forming aerial roots was substantially greater in the minicore than in the SAP, which underscores the potential of landraces for allele mining. The GWAS detected marker-trait associations with loci associated with transcription factors, phytohormone signaling, and root system architecture that we hypothesize to be involved in the formation of brace roots but that have a different expression profile in genotypes able to form aerial roots. In addition to a genetic contribution to aerial root formation, the environmental conditions during cultivation are essential, based on observed differences in aerial root phenotypes in Florida and Wisconsin. This is an important consideration for sorghum breeding programs aimed at incorporating this trait in advanced germplasm. Aerial root formation in sorghum shares several similarities with maize but also notable differences that make it premature to conclude that it is an ancestral trait.

## Supplementary Material

jkad285_Supplementary_Data

## Data Availability

The authors affirm that all data necessary for confirming the conclusions of the article are present within the article, figures, and tables. Supplemental material available at G3 online.
