# Supplementary material for: Identification of genetic and environmental factors influencing aerial root traits that support biological nitrogen fixation in sorghum
Source: G3 (Bethesda). 2023 Dec 14;14(3):jkad285. doi: 10.1093/g3journal/jkad285 (PMC10917507; doi:10.1093/g3journal/jkad285)
Supplement: jkad285_Supplementary_Data [file jkad285_supplementary_data.zip › Supplemental_Table_Legends_G3-2023-404694.pdf]

Legends for Supplemental Tables

Identification of genetic and environmental factors influencing aerial root traits that support biological nitrogen fixation in sorghum

Emily S. A. Wolf[1], Saddie Vela[1], Jennifer Wilker[2], Alyssa Davis[3], Madalen Robert[4,5], Valentina Infante[2], Rafael E. Venado[2], Cătălin Voiniciuc[5], Jean-Michel Ané[2,6], Wilfred Vermerris[1,3,7,*]

[1]Plant Molecular and Cellular Biology Graduate Program, University of Florida-Gainesville, FL 32609

[2]Department of Bacteriology, University of Wisconsin, Madison, WI 53706

[3]Department of Microbiology and Cell Science, University of Florida, Gainesville, FL 32610

[4]Independent Junior Research Group–Designer Glycans, Leibniz Institute of Plant Biochemistry, 06120 Halle (Saale), Germany

[5]Department of Horticultural Sciences, University of Florida, Gainesville, FL 32609

[6]Department of Agronomy, University of Wisconsin, Madison, WI 53706

[7]University of Florida Genetics Institute, University of Florida, Gainesville, FL32610

Supplemental Table 1. Complete phenotypic data set of the sorghum minicore and SAP for aerial root-related traits under standard and reduced (“low”) fertilizer applications in Florida and Wisconsin. Please see footnotes at the bottom of the table for details.

Supplemental Table 2. GWAS summary statistics of aerial root traits in sorghum under low fertilizer conditions.

Supplemental Table 3. List of genotypes evaluated in the GWAS with their corresponding SNP allele and phenotype.

Supplemental Table 4. SSR-based genotyping of 100 F_2 plants from two F_2 populations segregating for aerial root formation.

Supplemental Table 5. Primer sequences for sequencing and SSR-based genotyping

Supplemental Table 6. Relative composition of the monosaccharides present in sorghum aerial root mucilage of selected accessions cultivated under standard or low fertilizer conditions.
