# Supplementary material for: Identification of genetic and environmental factors influencing aerial root traits that support biological nitrogen fixation in sorghum
Source: G3 (Bethesda). 2023 Dec 14;14(3):jkad285. doi: 10.1093/g3journal/jkad285 (PMC10917507; doi:10.1093/g3journal/jkad285)
Supplement: jkad285_Supplementary_Data [file jkad285_supplementary_data.zip › Supplemental_Figures_G3-2023-404694.pdf]

Supplemental Figures

Identification of genetic and environmental factors influencing aerial root traits that support biological nitrogen fixation in sorghum

Emily S. A. Wolf[1], Saddie Vela[1], Jennifer Wilker[2], Alyssa Davis[3], Madalen Robert[4,5], Valentina Infante[2], Rafael E. Venado[2], Cătălin Voiniciuc[5], Jean-Michel Ané[2,6], Wilfred Vermerris[1,3,7,*]

[1]Plant Molecular and Cellular Biology Graduate Program, University of Florida-Gainesville, FL 32609

[2]Department of Bacteriology, University of Wisconsin, Madison, WI 53706

[3]Department of Microbiology and Cell Science, University of Florida, Gainesville, FL 32610

[4]Independent Junior Research Group–Designer Glycans, Leibniz Institute of Plant Biochemistry, 06120 Halle (Saale), Germany

[5]Department of Horticultural Sciences, University of Florida, Gainesville, FL 32609

[6]Department of Agronomy, University of Wisconsin, Madison, WI 53706

[7]University of Florida Genetics Institute, University of Florida, Gainesville, FL32610

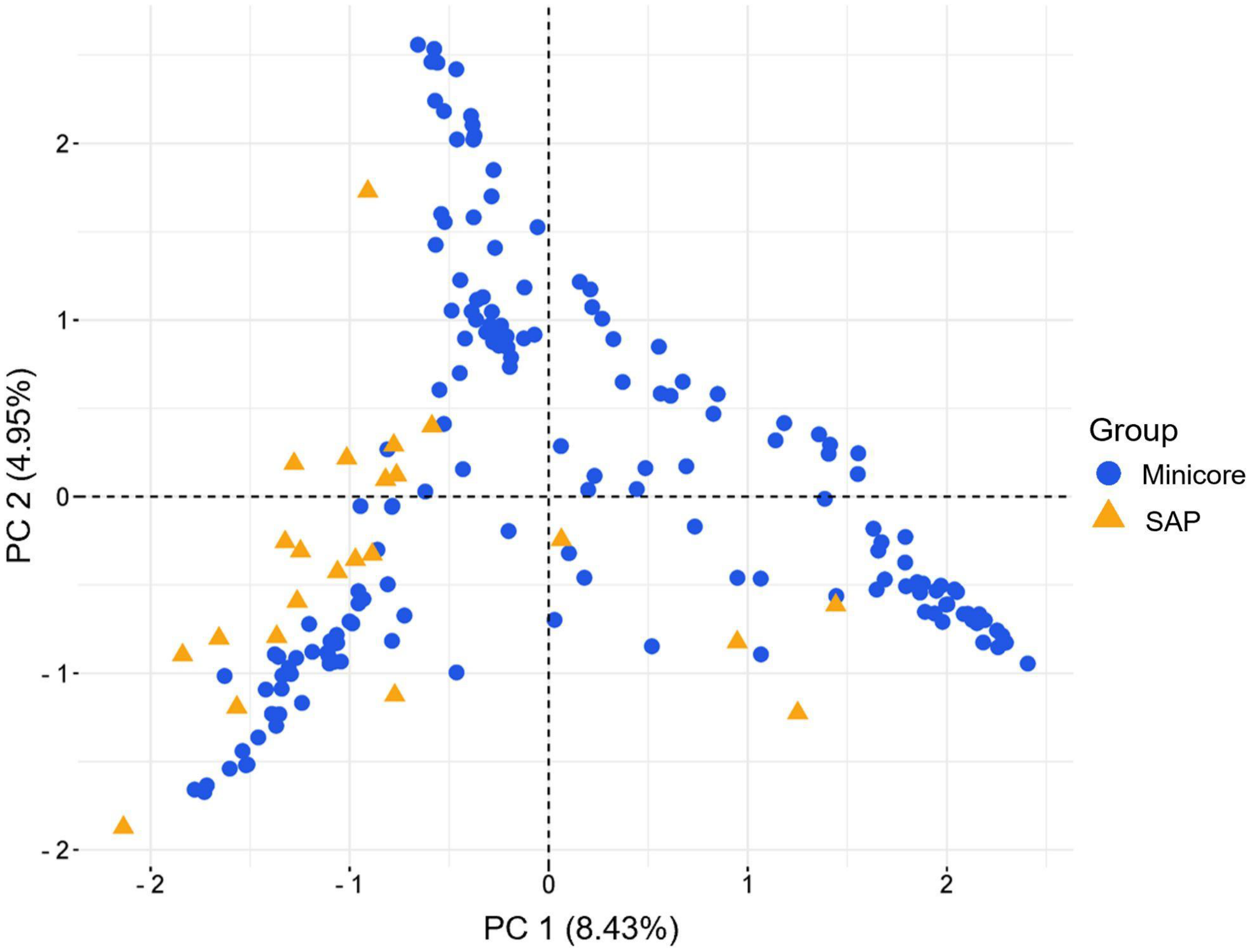

Supplemental Figure 1. Principal component score plot depicting the population structure of the sorghum minicore and SAP based on GBS data. Accessions from the minicore are represented by blue dots. The 30 selected accessions of the SAP are represented by orange triangles. The percentage of the variance explained by the two principal components are shown on their respective axes.

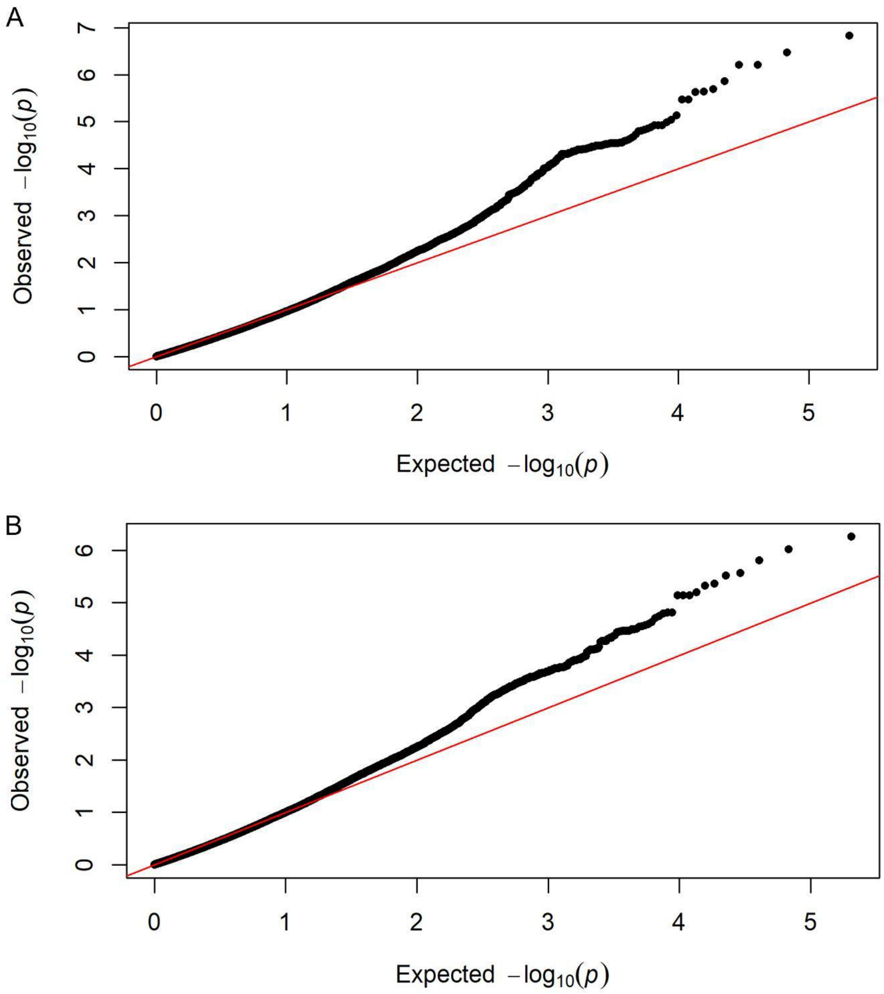

Supplemental Figure 2. Quantile-Quantile plots for the GWAS evaluating aerial root-related traits. The x-axis represents the expected -$\log_{10}$(P-values) and the y-axis the observed -$\log_{10}$(P-values). The red trend line represents the ideal Q-Q plot P-values. **A.** Q-Q plot for the GWAS for the number of nodes with aerial roots. **B.** Q-Q plot for the GWAS for aerial root diameter.

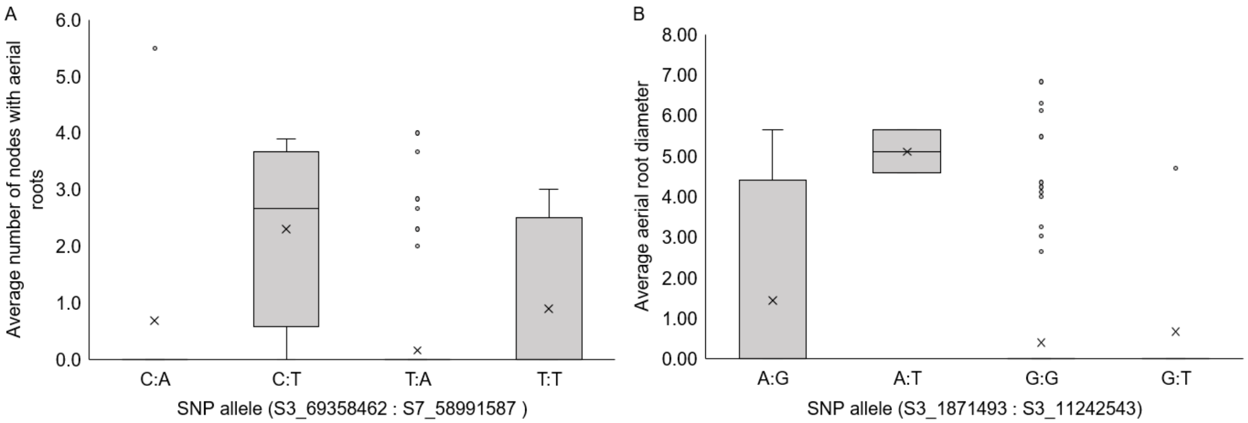

Supplemental Figure 3. Phenotypic distribution based on the SNP alleles detected in the GWAS. **A.** Boxplots displaying the average number of nodes with aerial roots as a function of the SNP alleles at the two loci S3_69358462 and S7_58991587. **B.** Boxplots displaying the average aerial root diameter as a function of the SNP alleles at the two loci S3_1871493 and S3_11242543). The box, line, whiskers, circles, and x, represent the interquartile range (when sufficient data points were available), the median, the minimum and maximum, outliers, and the average, respectively.

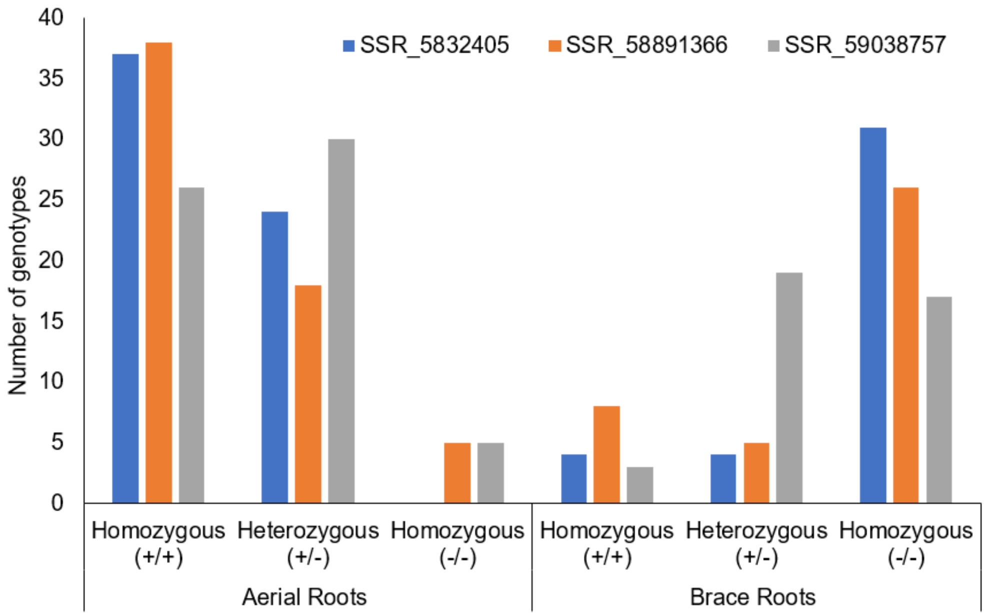

Supplemental Figure 4. Phenotypic distribution in two segregating F2 populations as a function of the SSR marker genotypes. The y-axis represents the number of individual plants in a given class, and the x-axis represents the marker genotypes at three SSR loci. The IS23992 allele is designated with a '+', and the UF15 and UF20 alleles with a '-'. The tallies at the loci SSR_5832405, SSR_58891366 and SSR_59038757 are displayed in blue, orange, and gray, respectively.

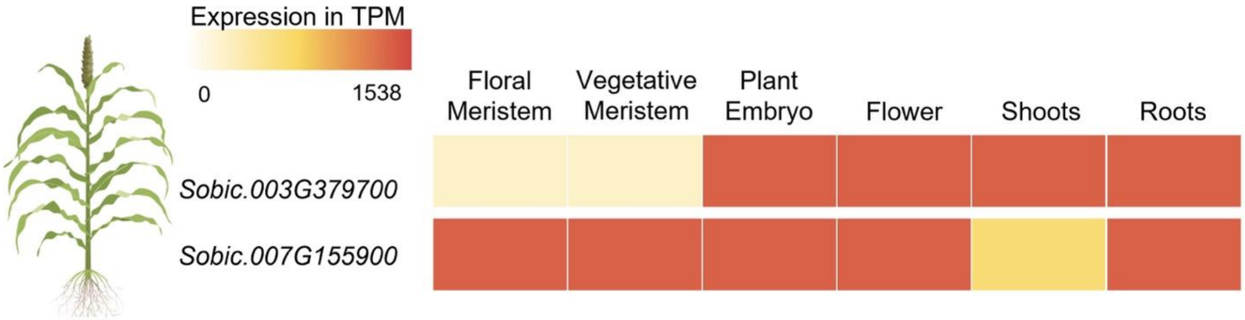

Supplemental Figure 5. Expression profile of candidate genes linked to the significant SNPs associated with the number of nodes with aerial roots in *Sorghum bicolor* (BTx623). The expression of each gene is displayed in normalized transcripts per million (TPM) for all tissues. TPM values are represented by a heatmap, ranging from light yellow to red, based on Olson et al. (2014). Shoot and root tissue was collected from seedlings that do not yet produce brace roots. Image created with BioRender.

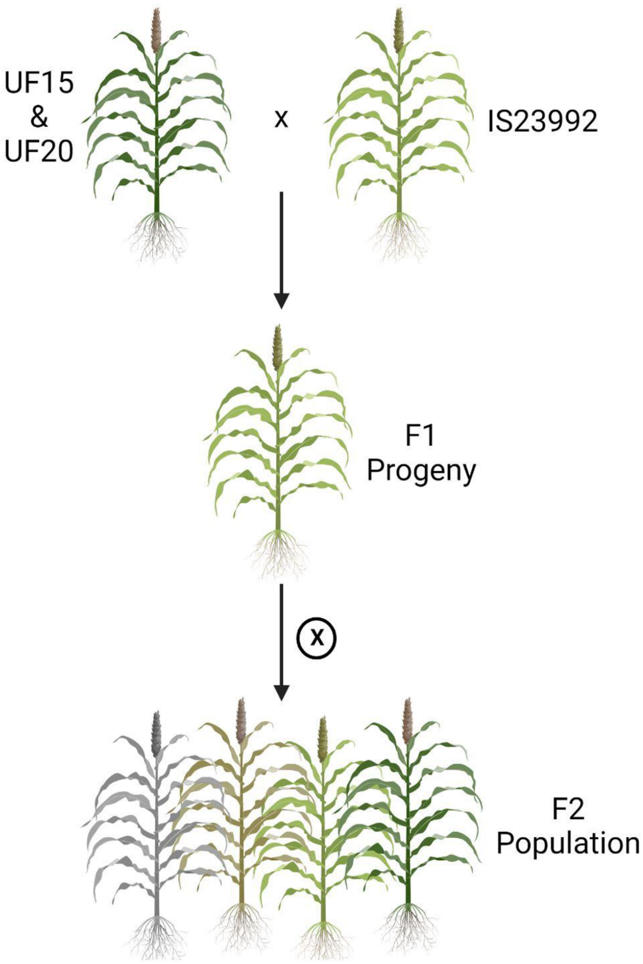

Supplemental Figure 6. Crossing scheme to develop two F_2 populations segregating for aerial root formation. The scheme represents a single population. F_1 progeny were selected and self-pollinated to generate an F_2 population segregating for aerial root formation.

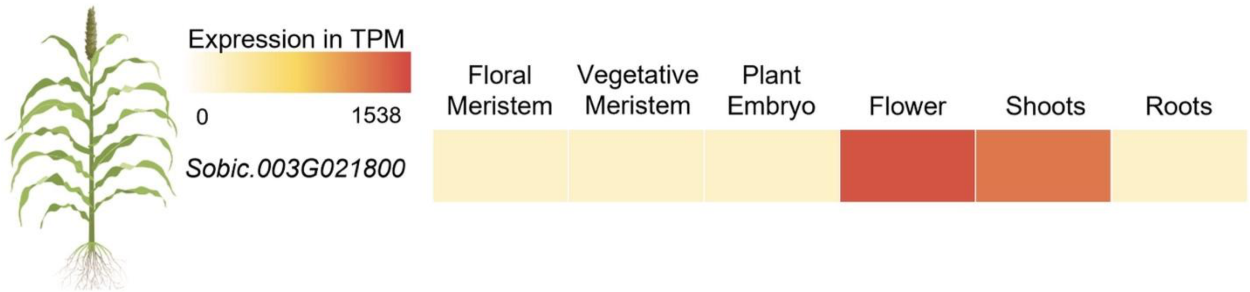

Supplemental Figure 7. Expression profile of candidate gene *Sobic.003G021800* associated with aerial root diameter in *Sorghum bicolor* (BTx623). The expression of *Sobic.003G021800* is shown in normalized transcripts per million (TPM) for all tissues. TPM values are represented by a heatmap, ranging from light yellow to red, based on Olson et al. (2014). Shoot and root tissue was collected from seedlings that do not yet produce brace roots. Image created with BioRender.

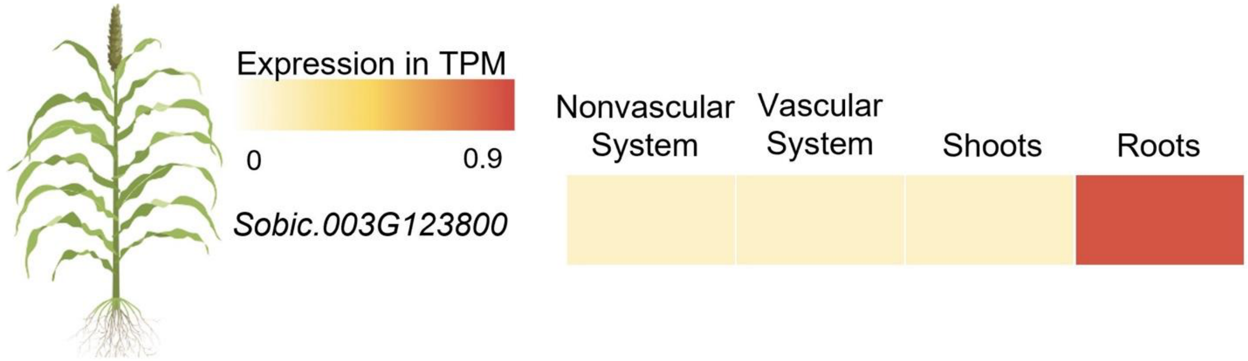

Supplemental Figure 8. The expression profile of candidate gene *Sobic.003G123800* is associated with aerial root diameter in *Sorghum bicolor* (BTx623). The expression of *Sobic.003G123800* is displayed in normalized transcripts per million (TPM). TPM values are represented by a heatmap, ranging from light yellow to red, based on Turco et al. (2017). Shoot and root tissue was collected from seedlings that do not yet produce brace roots. Image created with BioRender.

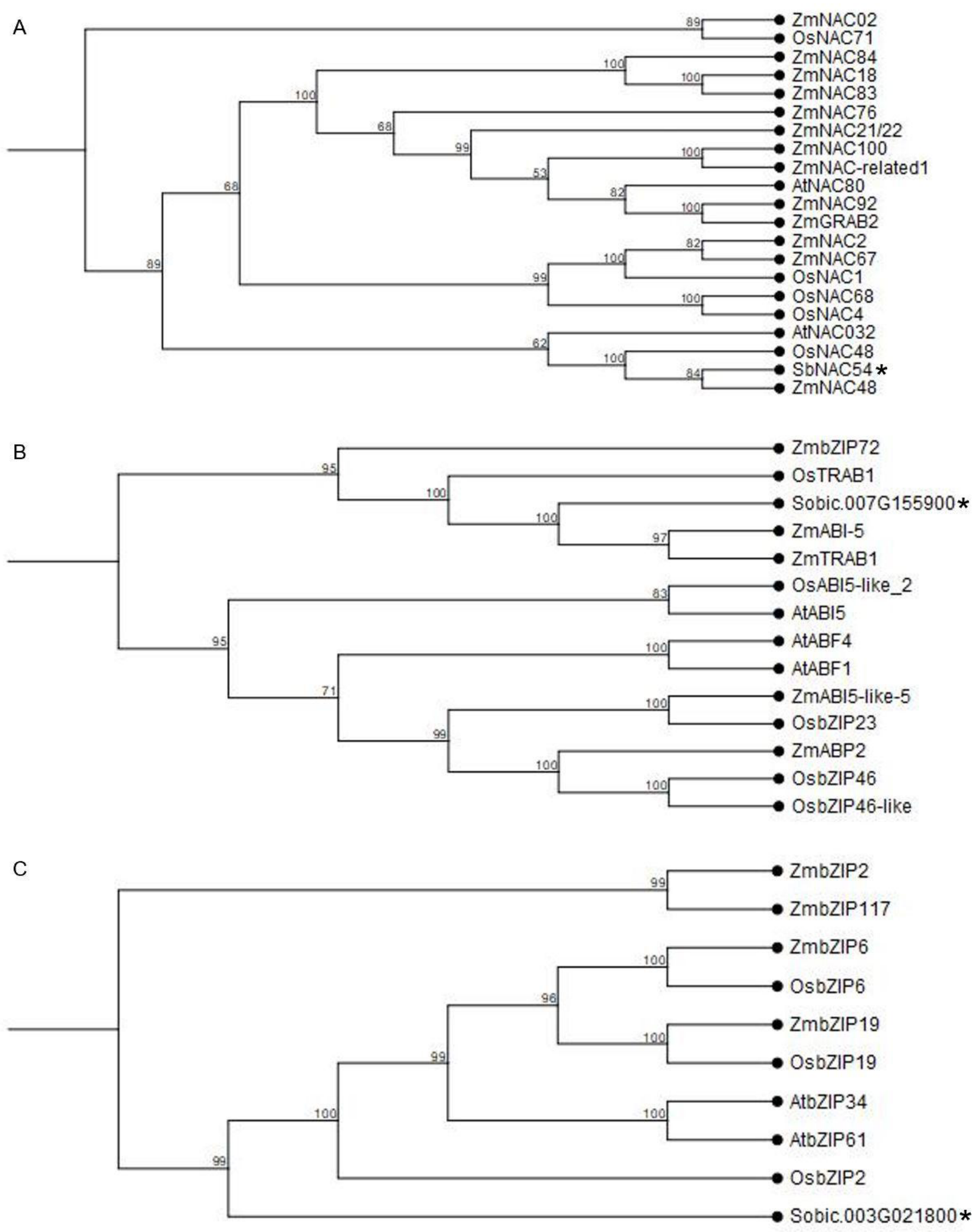

Supplemental Figure 9. Phylogenetic relationships inferred using maximum likelihood of predicted amino acid sequences encoded by sorghum, maize, rice, and Arabidopsis genes. Bootstrap values are displayed next to their respective node. Asterisks highlight the position of the proteins encoded by the sorghum gene. **A.** Phylogenetic tree of NAC transcription factors from the family Group I, subfamily ATAF that are predicted to be related to SbNAC54. **B.** Phylogenetic tree of bZIP transcription factors related to ABI5. **C.** Phylogenetic tree of the bZIP transcription factors related to Sobic.005G021800.

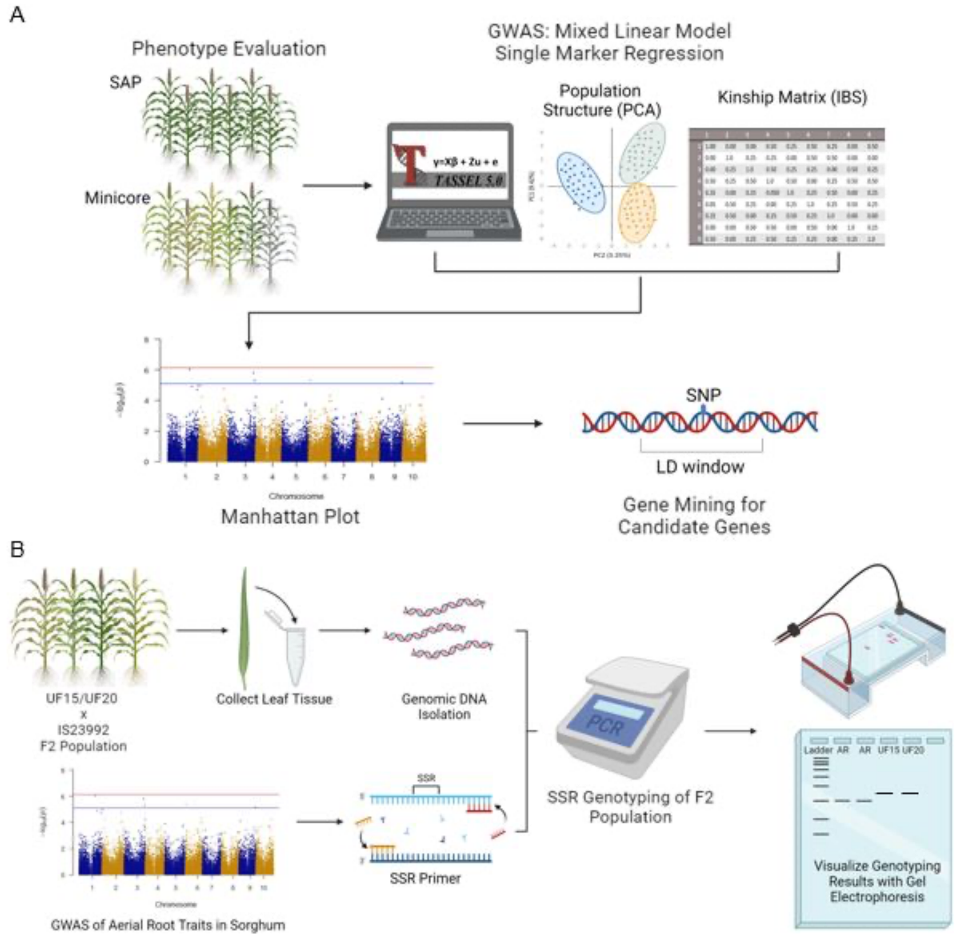

Supplemental Figure 10. Schematic representation of the methodologies used in this study. **A.** Pipeline employed to identify marker-trait associations for aerial root traits in sorghum. **B.** Methodology used to assess the pattern of inheritance of the aerial root phenotype in two F_2 populations using SSR markers flanking the SNPs identified in the GWAS. This figure was created with BioRender.

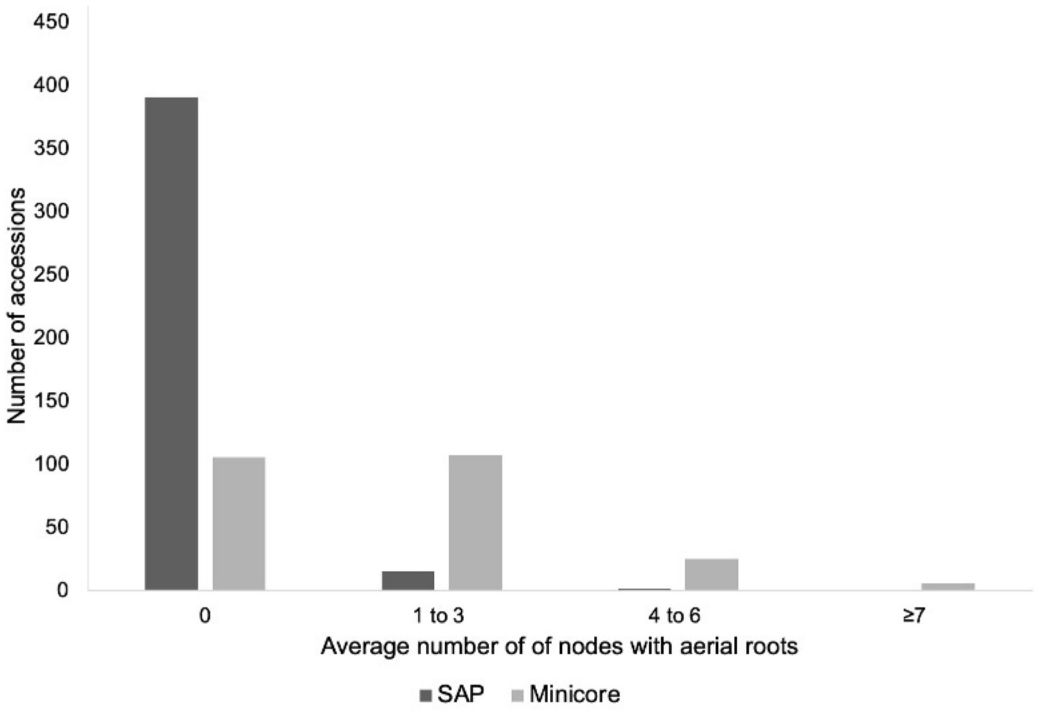

Supplemental Figure 11. Distribution of accessions in the SAP and minicore based on the average number of nodes with aerial roots.
